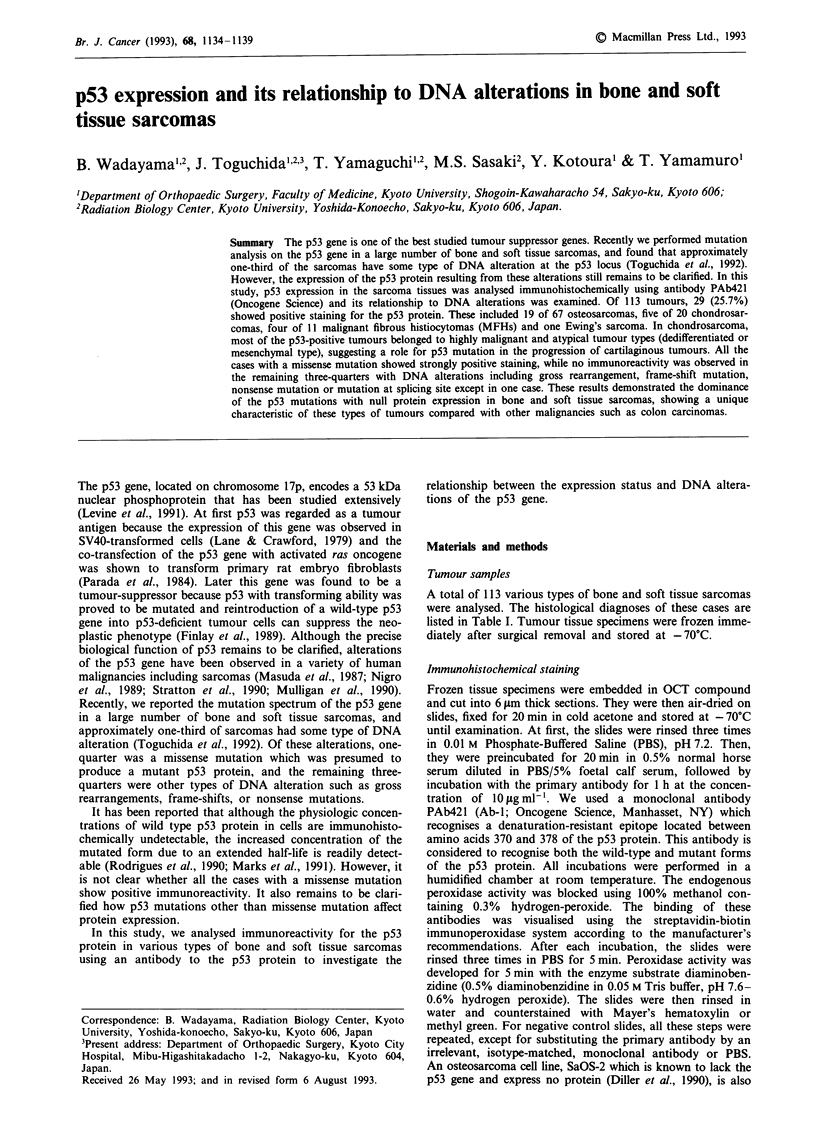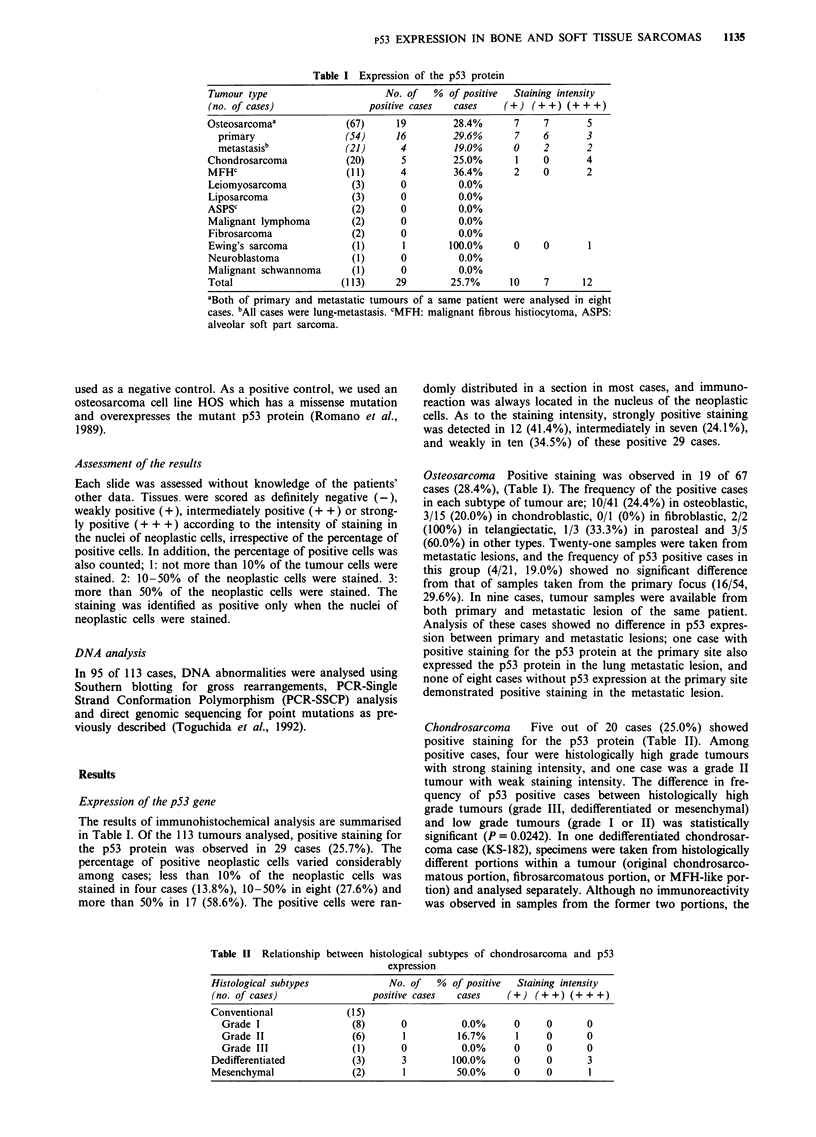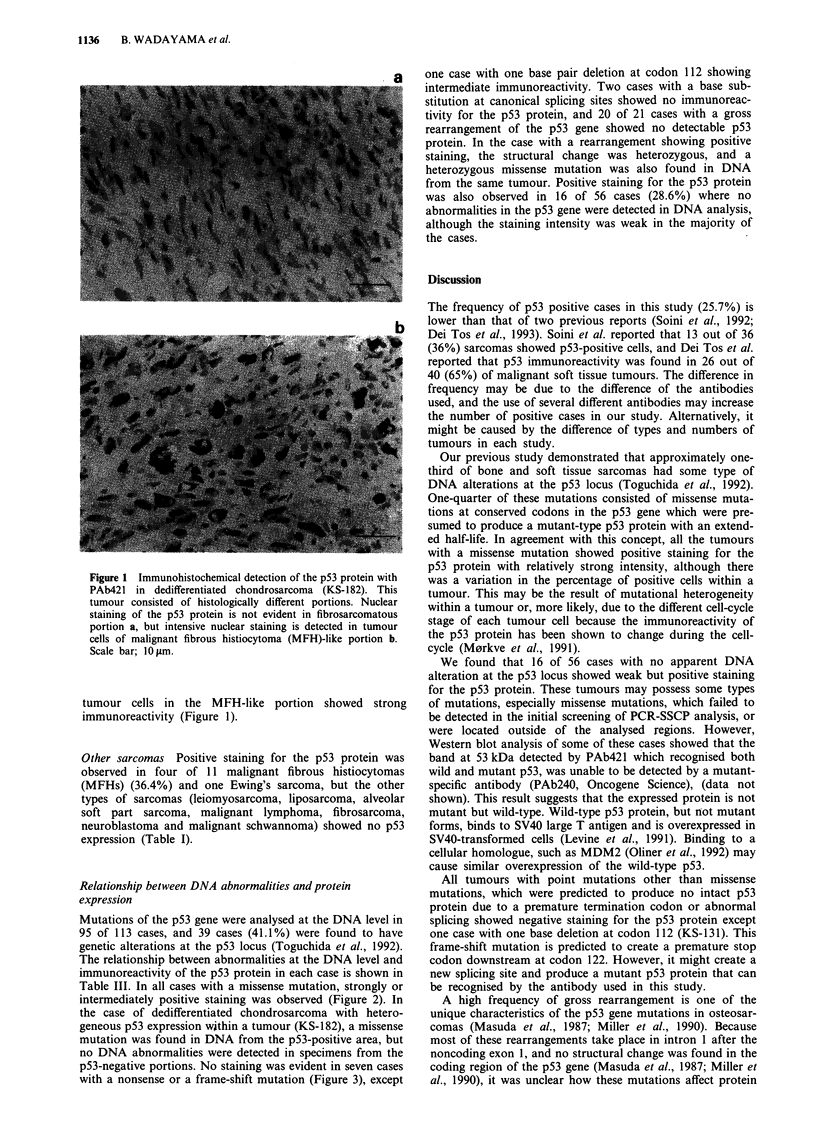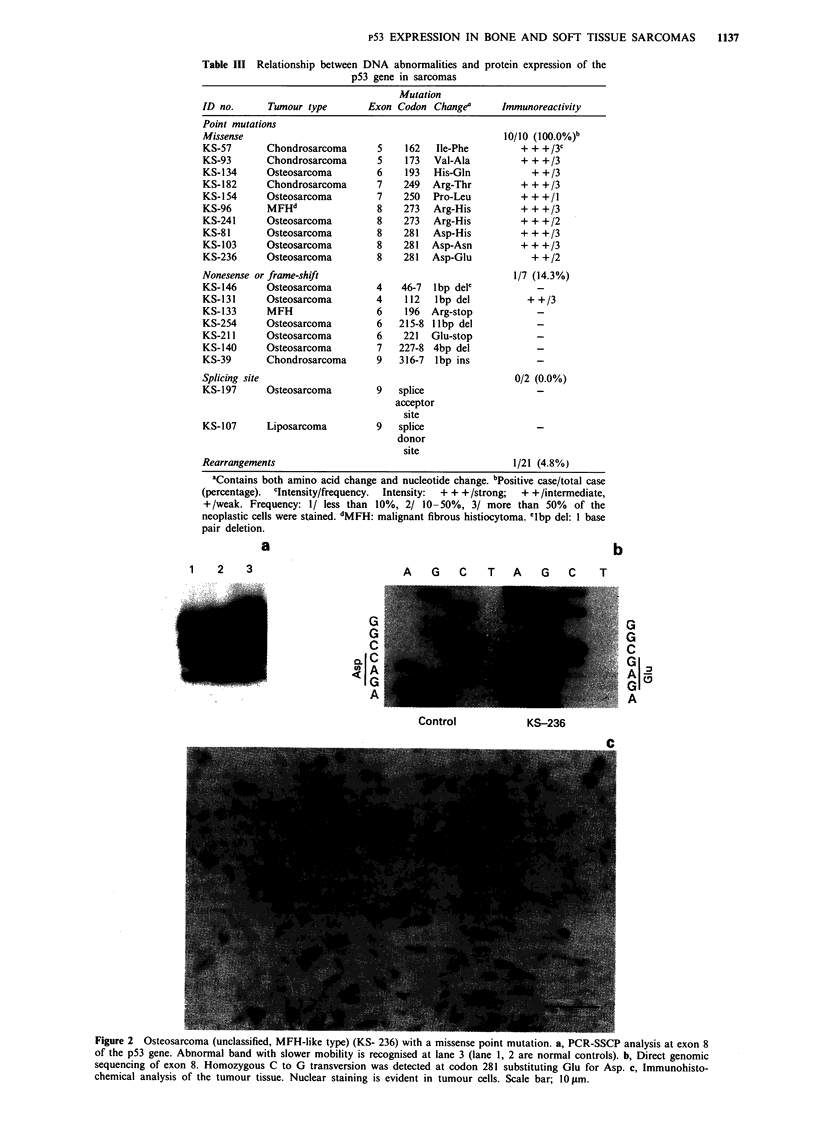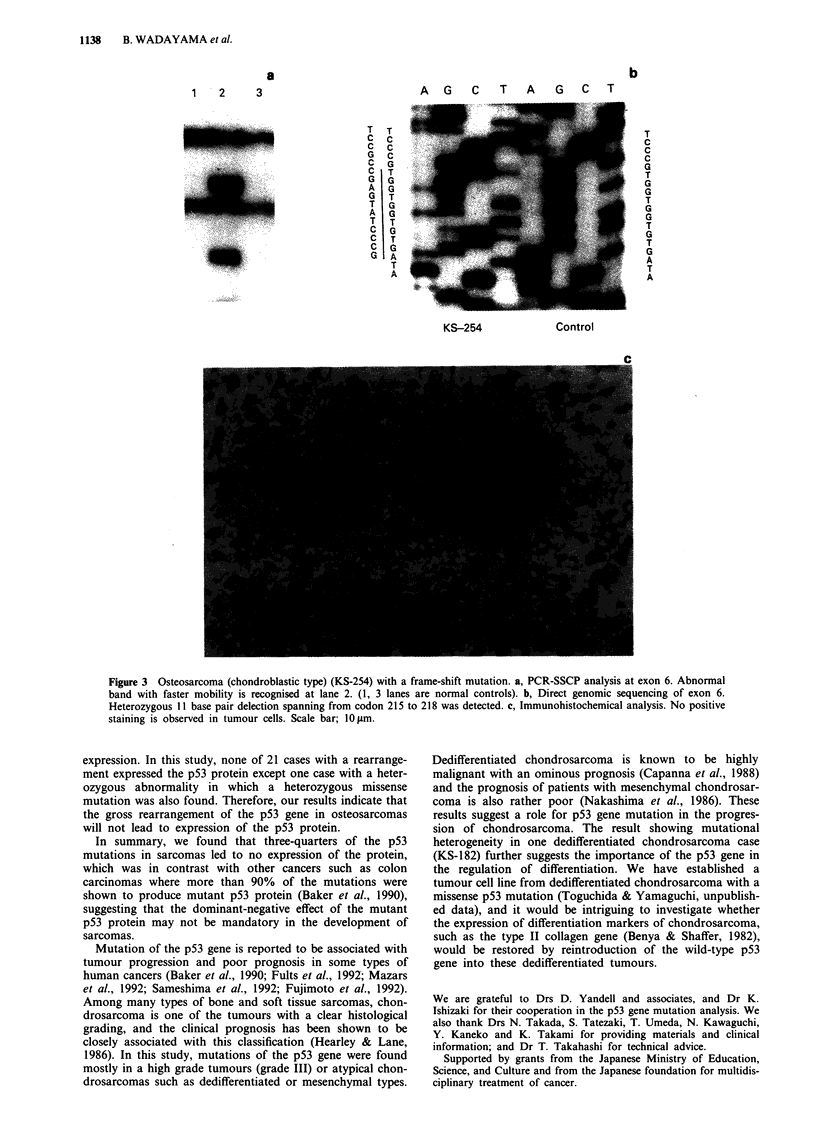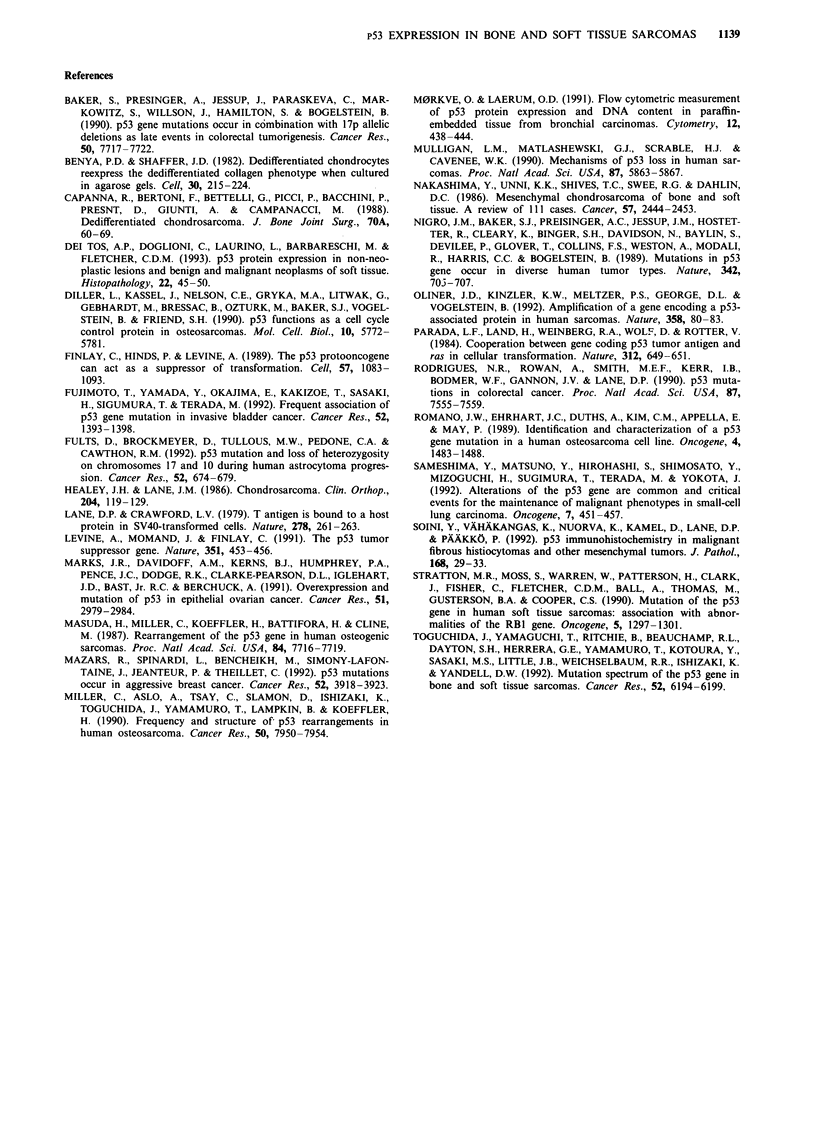# p53 expression and its relationship to DNA alterations in bone and soft tissue sarcomas.

**DOI:** 10.1038/bjc.1993.493

**Published:** 1993-12

**Authors:** B. Wadayama, J. Toguchida, T. Yamaguchi, M. S. Sasaki, T. Yamamuro

**Affiliations:** Department of Orthopaedic Surgery, Faculty of Medicine, Kyoto University, Japan.

## Abstract

**Images:**


Br. J. Cancer (1993), 68, 1134–1139 © Macmillan Press Ltd., 1993

### p53 expression and its relationship to DNA alterations in bone and soft tissue sarcomas

B. Wadayama[1,2], J. Toguchida[1,2,3], T. Yamaguchi[1,2], M.S. Sasaki[2], Y. Kotoura[1] & T. Yamamuro[1]

[1]Department of Orthopaedic Surgery, Faculty of Medicine, Kyoto University, Shogoin-Kawaharacho 54, Sakyo-ku, Kyoto 606; [2]Radiation Biology Center, Kyoto University, Yoshida-Konoecho, Sakyo-ku, Kyoto 606, Japan.

**Summary** The p53 gene is one of the best studied tumour suppressor genes. Recently we performed mutation analysis on the p53 gene in a large number of bone and soft tissue sarcomas, and found that approximately one-third of the sarcomas have some type of DNA alteration at the p53 locus (Toguchida *et al.*, 1992). However, the expression of the p53 protein resulting from these alterations still remains to be clarified. In this study, p53 expression in the sarcoma tissues was analysed immunohistochemically using antibody PAb421 (Oncogene Science) and its relationship to DNA alterations was examined. Of 113 tumours, 29 (25.7%) showed positive staining for the p53 protein. These included 19 of 67 osteosarcomas, five of 20 chondrosarcomas, four of 11 malignant fibrous histiocytomas (MFHs) and one Ewing's sarcoma. In chondrosarcoma, most of the p53-positive tumours belonged to highly malignant and atypical tumour types (dedifferentiated or mesenchymal type), suggesting a role for p53 mutation in the progression of cartilaginous tumours. All the cases with a missense mutation showed strongly positive staining, while no immunoreactivity was observed in the remaining three-quarters with DNA alterations including gross rearrangement, frame-shift mutation, nonsense mutation or mutation at splicing site except in one case. These results demonstrated the dominance of the p53 mutations with null protein expression in bone and soft tissue sarcomas, showing a unique characteristic of these types of tumours compared with other malignancies such as colon carcinomas.

The p53 gene, located on chromosome 17p, encodes a 53 kDa nuclear phosphoprotein that has been studied extensively (Levine *et al.*, 1991). At first p53 was regarded as a tumour antigen because the expression of this gene was observed in SV40-transformed cells (Lane & Crawford, 1979) and the co-transfection of the p53 gene with activated *ras* oncogene was shown to transform primary rat embryo fibroblasts (Parada *et al.*, 1984). Later this gene was found to be a tumour-suppressor because p53 with transforming ability was proved to be mutated and reintroduction of a wild-type p53 gene into p53-deficient tumour cells can suppress the neoplastic phenotype (Finlay *et al.*, 1989). Although the precise biological function of p53 remains to be clarified, alterations of the p53 gene have been observed in a variety of human malignancies including sarcomas (Masuda *et al.*, 1987; Nigro *et al.*, 1989; Stratton *et al.*, 1990; Mulligan *et al.*, 1990). Recently, we reported the mutation spectrum of the p53 gene in a large number of bone and soft tissue sarcomas, and approximately one-third of sarcomas had some type of DNA alteration (Toguchida *et al.*, 1992). Of these alterations, one-quarter was a missense mutation which was presumed to produce a mutant p53 protein, and the remaining three-quarters were other types of DNA alteration such as gross rearrangements, frame-shifts, or nonsense mutations.

It has been reported that although the physiologic concentrations of wild type p53 protein in cells are immunohistochemically undetectable, the increased concentration of the mutated form due to an extended half-life is readily detectable (Rodrigues *et al.*, 1990; Marks *et al.*, 1991). However, it is not clear whether all the cases with a missense mutation show positive immunoreactivity. It also remains to be clarified how p53 mutations other than missense mutation affect protein expression.

In this study, we analysed immunoreactivity for the p53 protein in various types of bone and soft tissue sarcomas using an antibody to the p53 protein to investigate the relationship between the expression status and DNA alterations of the p53 gene.

### Materials and methods

### *Tumour samples*

A total of 113 various types of bone and soft tissue sarcomas were analysed. The histological diagnoses of these cases are listed in Table I. Tumour tissue specimens were frozen immediately after surgical removal and stored at − 70°C.

### *Immunohistochemical staining*

Frozen tissue specimens were embedded in OCT compound and cut into 6 μm thick sections. They were then air-dried on slides, fixed for 20 min in cold acetone and stored at − 70°C until examination. At first, the slides were rinsed three times in 0.01 M Phosphate-Buffered Saline (PBS), pH 7.2. Then, they were preincubated for 20 min in 0.5% normal horse serum diluted in PBS/5% foetal calf serum, followed by incubation with the primary antibody for 1 h at the concentration of 10 $\mu g\ ml^{-1}$. We used a monoclonal antibody PAb421 (Ab-1; Oncogene Science, Manhasset, NY) which recognises a denaturation-resistant epitope located between amino acids 370 and 378 of the p53 protein. This antibody is considered to recognise both the wild-type and mutant forms of the p53 protein. All incubations were performed in a humidified chamber at room temperature. The endogenous peroxidase activity was blocked using 100% methanol containing 0.3% hydrogen-peroxide. The binding of these antibodies was visualised using the streptavidin-biotin immunoperoxidase system according to the manufacturer's recommendations. After each incubation, the slides were rinsed three times in PBS for 5 min. Peroxidase activity was developed for 5 min with the enzyme substrate diaminobenzidine (0.5% diaminobenzidine in 0.05 M Tris buffer, pH 7.6–0.6% hydrogen peroxide). The slides were then rinsed in water and counterstained with Mayer's hematoxylin or methyl green. For negative control slides, all these steps were repeated, except for substituting the primary antibody by an irrelevant, isotype-matched, monoclonal antibody or PBS. An osteosarcoma cell line, SaOS-2 which is known to lack the p53 gene and express no protein (Diller *et al.*, 1990), is also

Correspondence: B. Wadayama, Radiation Biology Center, Kyoto University, Yoshida-konoecho, Sakyo-ku, Kyoto 606, Japan
[3]Present address: Department of Orthopaedic Surgery, Kyoto City Hospital, Mibu-Higashitakadacho 1-2, Nakagyo-ku, Kyoto 604, Japan.
Received 26 May 1993; and in revised form 6 August 1993.

**Table I** Expression of the p53 protein

| *Tumour type (no. of cases)* | | *No. of positive cases* | *% of positive cases* | *Staining intensity (+)* | *(++)* | *(+++)* |
|---|---|---|---|---|---|---|
| Osteosarcoma[a] | (67) | 19 | 28.4% | 7 | 7 | 5 |
| primary | *(54)* | *16* | *29.6%* | *7* | *6* | *3* |
| metastasis[b] | *(21)* | *4* | *19.0%* | *0* | *2* | *2* |
| Chondrosarcoma | (20) | 5 | 25.0% | 1 | 0 | 4 |
| MFH[c] | (11) | 4 | 36.4% | 2 | 0 | 2 |
| Leiomyosarcoma | (3) | 0 | 0.0% | | | |
| Liposarcoma | (3) | 0 | 0.0% | | | |
| ASPS[c] | (2) | 0 | 0.0% | | | |
| Malignant lymphoma | (2) | 0 | 0.0% | | | |
| Fibrosarcoma | (2) | 0 | 0.0% | | | |
| Ewing's sarcoma | (1) | 1 | 100.0% | 0 | 0 | 1 |
| Neuroblastoma | (1) | 0 | 0.0% | | | |
| Malignant schwannoma | (1) | 0 | 0.0% | | | |
| Total | (113) | 29 | 25.7% | 10 | 7 | 12 |

[a]Both of primary and metastatic tumours of a same patient were analysed in eight cases. [b]All cases were lung-metastasis. [c]MFH: malignant fibrous histiocytoma, ASPS: alveolar soft part sarcoma.

used as a negative control. As a positive control, we used an osteosarcoma cell line HOS which has a missense mutation and overexpresses the mutant p53 protein (Romano *et al.*, 1989).

### *Assessment of the results*

Each slide was assessed without knowledge of the patients' other data. Tissues were scored as definitely negative (−), weakly positive (+), intermediately positive (+ +) or strongly positive (+ + +) according to the intensity of staining in the nuclei of neoplastic cells, irrespective of the percentage of positive cells. In addition, the percentage of positive cells was also counted; 1: not more than 10% of the tumour cells were stained. 2: 10–50% of the neoplastic cells were stained. 3: more than 50% of the neoplastic cells were stained. The staining was identified as positive only when the nuclei of neoplastic cells were stained.

### *DNA analysis*

In 95 of 113 cases, DNA abnormalities were analysed using Southern blotting for gross rearrangements, PCR-Single Strand Conformation Polymorphism (PCR-SSCP) analysis and direct genomic sequencing for point mutations as previously described (Toguchida *et al.*, 1992).

### Results

### *Expression of the p53 gene*

The results of immunohistochemical analysis are summarised in Table I. Of the 113 tumours analysed, positive staining for the p53 protein was observed in 29 cases (25.7%). The percentage of positive neoplastic cells varied considerably among cases; less than 10% of the neoplastic cells was stained in four cases (13.8%), 10–50% in eight (27.6%) and more than 50% in 17 (58.6%). The positive cells were randomly distributed in a section in most cases, and immunoreaction was always located in the nucleus of the neoplastic cells. As to the staining intensity, strongly positive staining was detected in 12 (41.4%), intermediately in seven (24.1%), and weakly in ten (34.5%) of these positive 29 cases.

*Osteosarcoma* Positive staining was observed in 19 of 67 cases (28.4%), (Table I). The frequency of the positive cases in each subtype of tumour are; 10/41 (24.4%) in osteoblastic, 3/15 (20.0%) in chondroblastic, 0/1 (0%) in fibroblastic, 2/2 (100%) in telangiectatic, 1/3 (33.3%) in parosteal and 3/5 (60.0%) in other types. Twenty-one samples were taken from metastatic lesions, and the frequency of p53 positive cases in this group (4/21, 19.0%) showed no significant difference from that of samples taken from the primary focus (16/54, 29.6%). In nine cases, tumour samples were available from both primary and metastatic lesion of the same patient. Analysis of these cases showed no difference in p53 expression between primary and metastatic lesions; one case with positive staining for the p53 protein at the primary site also expressed the p53 protein in the lung metastatic lesion, and none of eight cases without p53 expression at the primary site demonstrated positive staining in the metastatic lesion.

*Chondrosarcoma* Five out of 20 cases (25.0%) showed positive staining for the p53 protein (Table II). Among positive cases, four were histologically high grade tumours with strong staining intensity, and one case was a grade II tumour with weak staining intensity. The difference in frequency of p53 positive cases between histologically high grade tumours (grade III, dedifferentiated or mesenchymal) and low grade tumours (grade I or II) was statistically significant ($P = 0.0242$). In one dedifferentiated chondrosarcoma case (KS-182), specimens were taken from histologically different portions within a tumour (original chondrosarcomatous portion, fibrosarcomatous portion, or MFH-like portion) and analysed separately. Although no immunoreactivity was observed in samples from the former two portions, the

**Table II** Relationship between histological subtypes of chondrosarcoma and p53 expression

| *Histological subtypes (no. of cases)* | | *No. of positive cases* | *% of positive cases* | *Staining intensity (+)* | *(++)* | *(+++)* |
|---|---|---|---|---|---|---|
| Conventional | (15) | | | | | |
| Grade I | (8) | 0 | 0.0% | 0 | 0 | 0 |
| Grade II | (6) | 1 | 16.7% | 1 | 0 | 0 |
| Grade III | (1) | 0 | 0.0% | 0 | 0 | 0 |
| Dedifferentiated | (3) | 3 | 100.0% | 0 | 0 | 3 |
| Mesenchymal | (2) | 1 | 50.0% | 0 | 0 | 1 |

**Figure 1** Immunohistochemical detection of the p53 protein with PAb421 in dedifferentiated chondrosarcoma (KS-182). This tumour consisted of histologically different portions. Nuclear staining of the p53 protein is not evident in fibrosarcomatous portion **a**, but intensive nuclear staining is detected in tumour cells of malignant fibrous histiocytoma (MFH)-like portion **b**. Scale bar; 10 µm.

tumour cells in the MFH-like portion showed strong immunoreactivity (Figure 1).

*Other sarcomas* Positive staining for the p53 protein was observed in four of 11 malignant fibrous histiocytomas (MFHs) (36.4%) and one Ewing's sarcoma, but the other types of sarcomas (leiomyosarcoma, liposarcoma, alveolar soft part sarcoma, malignant lymphoma, fibrosarcoma, neuroblastoma and malignant schwannoma) showed no p53 expression (Table I).

*Relationship between DNA abnormalities and protein expression*

Mutations of the p53 gene were analysed at the DNA level in 95 of 113 cases, and 39 cases (41.1%) were found to have genetic alterations at the p53 locus (Toguchida *et al.*, 1992). The relationship between abnormalities at the DNA level and immunoreactivity of the p53 protein in each case is shown in Table III. In all cases with a missense mutation, strongly or intermediately positive staining was observed (Figure 2). In the case of dedifferentiated chondrosarcoma with heterogeneous p53 expression within a tumour (KS-182), a missense mutation was found in DNA from the p53-positive area, but no DNA abnormalities were detected in specimens from the p53-negative portions. No staining was evident in seven cases with a nonsense or a frame-shift mutation (Figure 3), except one case with one base pair deletion at codon 112 showing intermediate immunoreactivity. Two cases with a base substitution at canonical splicing sites showed no immunoreactivity for the p53 protein, and 20 of 21 cases with a gross rearrangement of the p53 gene showed no detectable p53 protein. In the case with a rearrangement showing positive staining, the structural change was heterozygous, and a heterozygous missense mutation was also found in DNA from the same tumour. Positive staining for the p53 protein was also observed in 16 of 56 cases (28.6%) where no abnormalities in the p53 gene were detected in DNA analysis, although the staining intensity was weak in the majority of the cases.

### Discussion

The frequency of p53 positive cases in this study (25.7%) is lower than that of two previous reports (Soini *et al.*, 1992; Dei Tos *et al.*, 1993). Soini *et al.* reported that 13 out of 36 (36%) sarcomas showed p53-positive cells, and Dei Tos *et al.* reported that p53 immunoreactivity was found in 26 out of 40 (65%) of malignant soft tissue tumours. The difference in frequency may be due to the difference of the antibodies used, and the use of several different antibodies may increase the number of positive cases in our study. Alternatively, it might be caused by the difference of types and numbers of tumours in each study.

Our previous study demonstrated that approximately one-third of bone and soft tissue sarcomas had some type of DNA alterations at the p53 locus (Toguchida *et al.*, 1992). One-quarter of these mutations consisted of missense mutations at conserved codons in the p53 gene which were presumed to produce a mutant-type p53 protein with an extended half-life. In agreement with this concept, all the tumours with a missense mutation showed positive staining for the p53 protein with relatively strong intensity, although there was a variation in the percentage of positive cells within a tumour. This may be the result of mutational heterogeneity within a tumour or, more likely, due to the different cell-cycle stage of each tumour cell because the immunoreactivity of the p53 protein has been shown to change during the cell-cycle (Mørkve *et al.*, 1991).

We found that 16 of 56 cases with no apparent DNA alteration at the p53 locus showed weak but positive staining for the p53 protein. These tumours may possess some types of mutations, especially missense mutations, which failed to be detected in the initial screening of PCR-SSCP analysis, or were located outside of the analysed regions. However, Western blot analysis of some of these cases showed that the band at 53 kDa detected by PAb421 which recognised both wild and mutant p53, was unable to be detected by a mutant-specific antibody (PAb240, Oncogene Science), (data not shown). This result suggests that the expressed protein is not mutant but wild-type. Wild-type p53 protein, but not mutant forms, binds to SV40 large T antigen and is overexpressed in SV40-transformed cells (Levine *et al.*, 1991). Binding to a cellular homologue, such as MDM2 (Oliner *et al.*, 1992) may cause similar overexpression of the wild-type p53.

All tumours with point mutations other than missense mutations, which were predicted to produce no intact p53 protein due to a premature termination codon or abnormal splicing showed negative staining for the p53 protein except one case with one base deletion at codon 112 (KS-131). This frame-shift mutation is predicted to create a premature stop codon downstream at codon 122. However, it might create a new splicing site and produce a mutant p53 protein that can be recognised by the antibody used in this study.

A high frequency of gross rearrangement is one of the unique characteristics of the p53 gene mutations in osteosarcomas (Masuda *et al.*, 1987; Miller *et al.*, 1990). Because most of these rearrangements take place in intron 1 after the noncoding exon 1, and no structural change was found in the coding region of the p53 gene (Masuda *et al.*, 1987; Miller *et al.*, 1990), it was unclear how these mutations affect protein

**Table III** Relationship between DNA abnormalities and protein expression of the p53 gene in sarcomas

| *ID no.* | *Tumour type* | *Exon* | *Codon* | *Change*[a] | *Immunoreactivity* |
|---|---|---|---|---|---|
| *Point mutations* | | | | | |
| *Missense* | | | | | 10/10 (100.0%)[b] |
| KS-57 | Chondrosarcoma | 5 | 162 | Ile-Phe | + + +/3[c] |
| KS-93 | Chondrosarcoma | 5 | 173 | Val-Ala | + + +/3 |
| KS-134 | Osteosarcoma | 6 | 193 | His-Gln | + +/3 |
| KS-182 | Chondrosarcoma | 7 | 249 | Arg-Thr | + + +/3 |
| KS-154 | Osteosarcoma | 7 | 250 | Pro-Leu | + + +/1 |
| KS-96 | MFH[d] | 8 | 273 | Arg-His | + + +/3 |
| KS-241 | Osteosarcoma | 8 | 273 | Arg-His | + + +/2 |
| KS-81 | Osteosarcoma | 8 | 281 | Asp-His | + + +/3 |
| KS-103 | Osteosarcoma | 8 | 281 | Asp-Asn | + + +/3 |
| KS-236 | Osteosarcoma | 8 | 281 | Asp-Glu | + +/2 |
| *Nonesense or frame-shift* | | | | | 1/7 (14.3%) |
| KS-146 | Osteosarcoma | 4 | 46-7 | 1bp del[e] | – |
| KS-131 | Osteosarcoma | 4 | 112 | 1bp del | + +/3 |
| KS-133 | MFH | 6 | 196 | Arg-stop | – |
| KS-254 | Osteosarcoma | 6 | 215-8 | 11bp del | – |
| KS-211 | Osteosarcoma | 6 | 221 | Glu-stop | – |
| KS-140 | Osteosarcoma | 7 | 227-8 | 4bp del | – |
| KS-39 | Chondrosarcoma | 9 | 316-7 | 1bp ins | – |
| *Splicing site* | | | | | 0/2 (0.0%) |
| KS-197 | Osteosarcoma | 9 | splice acceptor site | | – |
| KS-107 | Liposarcoma | 9 | splice donor site | | – |
| *Rearrangements* | | | | | 1/21 (4.8%) |

(*Mutation* spans Exon, Codon, Change.)

[a]Contains both amino acid change and nucleotide change. [b]Positive case/total case (percentage). [c]Intensity/frequency. Intensity: + + +/strong; + +/intermediate, +/weak. Frequency: 1/ less than 10%, 2/ 10–50%, 3/ more than 50% of the neoplastic cells were stained. [d]MFH: malignant fibrous histiocytoma. [e]1bp del: 1 base pair deletion.

**Figure 2** Osteosarcoma (unclassified, MFH-like type) (KS- 236) with a missense point mutation. **a**, PCR-SSCP analysis at exon 8 of the p53 gene. Abnormal band with slower mobility is recognised at lane 3 (lane 1, 2 are normal controls). **b**, Direct genomic sequencing of exon 8. Homozygous C to G transversion was detected at codon 281 substituting Glu for Asp. **c**, Immunohistochemical analysis of the tumour tissue. Nuclear staining is evident in tumour cells. Scale bar; 10 μm.

**Figure 3** Osteosarcoma (chondroblastic type) (KS-254) with a frame-shift mutation. **a**, PCR-SSCP analysis at exon 6. Abnormal band with faster mobility is recognised at lane 2. (1, 3 lanes are normal controls). **b**, Direct genomic sequencing of exon 6. Heterozygous 11 base pair delection spanning from codon 215 to 218 was detected. **c**, Immunohistochemical analysis. No positive staining is observed in tumour cells. Scale bar; 10 $\mu$m.

expression. In this study, none of 21 cases with a rearrangement expressed the p53 protein except one case with a heterozygous abnormality in which a heterozygous missense mutation was also found. Therefore, our results indicate that the gross rearrangement of the p53 gene in osteosarcomas will not lead to expression of the p53 protein.

In summary, we found that three-quarters of the p53 mutations in sarcomas led to no expression of the protein, which was in contrast with other cancers such as colon carcinomas where more than 90% of the mutations were shown to produce mutant p53 protein (Baker *et al.*, 1990), suggesting that the dominant-negative effect of the mutant p53 protein may not be mandatory in the development of sarcomas.

Mutation of the p53 gene is reported to be associated with tumour progression and poor prognosis in some types of human cancers (Baker *et al.*, 1990; Fults *et al.*, 1992; Mazars *et al.*, 1992; Sameshima *et al.*, 1992; Fujimoto *et al.*, 1992). Among many types of bone and soft tissue sarcomas, chondrosarcoma is one of the tumours with a clear histological grading, and the clinical prognosis has been shown to be closely associated with this classification (Hearley & Lane, 1986). In this study, mutations of the p53 gene were found mostly in a high grade tumours (grade III) or atypical chondrosarcomas such as dedifferentiated or mesenchymal types. Dedifferentiated chondrosarcoma is known to be highly malignant with an ominous prognosis (Capanna *et al.*, 1988) and the prognosis of patients with mesenchymal chondrosarcoma is also rather poor (Nakashima *et al.*, 1986). These results suggest a role for p53 gene mutation in the progression of chondrosarcoma. The result showing mutational heterogeneity in one dedifferentiated chondrosarcoma case (KS-182) further suggests the importance of the p53 gene in the regulation of differentiation. We have established a tumour cell line from dedifferentiated chondrosarcoma with a missense p53 mutation (Toguchida & Yamaguchi, unpublished data), and it would be intriguing to investigate whether the expression of differentiation markers of chondrosarcoma, such as the type II collagen gene (Benya & Shaffer, 1982), would be restored by reintroduction of the wild-type p53 gene into these dedifferentiated tumours.

We are grateful to Drs D. Yandell and associates, and Dr K. Ishizaki for their cooperation in the p53 gene mutation analysis. We also thank Drs N. Takada, S. Tatezaki, T. Umeda, N. Kawaguchi, Y. Kaneko and K. Takami for providing materials and clinical information; and Dr T. Takahashi for technical advice.

Supported by grants from the Japanese Ministry of Education, Science, and Culture and from the Japanese foundation for multidisciplinary treatment of cancer.

### References

BAKER, S., PRESINGER, A., JESSUP, J., PARASKEVA, C., MARKOWITZ, S., WILLSON, J., HAMILTON, S. & BOGELSTEIN, B. (1990). p53 gene mutations occur in combination with 17p allelic deletions as late events in colorectal tumorigenesis. *Cancer Res.*, **50**, 7717–7722.

BENYA, P.D. & SHAFFER, J.D. (1982). Dedifferentiated chondrocytes reexpress the dedifferentiated collagen phenotype when cultured in agarose gels. *Cell*, **30**, 215–224.

CAPANNA, R., BERTONI, F., BETTELLI, G., PICCI, P., BACCHINI, P., PRESNT, D., GIUNTI, A. & CAMPANACCI, M. (1988). Dedifferentiated chondrosarcoma. *J. Bone Joint Surg.*, **70A**, 60–69.

DEI TOS, A.P., DOGLIONI, C., LAURINO, L., BARBARESCHI, M. & FLETCHER, C.D.M. (1993). p53 protein expression in non-neoplastic lesions and benign and malignant neoplasms of soft tissue. *Histopathology*, **22**, 45–50.

DILLER, L., KASSEL, J., NELSON, C.E., GRYKA, M.A., LITWAK, G., GEBHARDT, M., BRESSAC, B., OZTURK, M., BAKER, S.J., VOGELSTEIN, B. & FRIEND, S.H. (1990). p53 functions as a cell cycle control protein in osteosarcomas. *Mol. Cell. Biol.*, **10**, 5772–5781.

FINLAY, C., HINDS, P. & LEVINE, A. (1989). The p53 protooncogene can act as a suppressor of transformation. *Cell*, **57**, 1083–1093.

FUJIMOTO, T., YAMADA, Y., OKAJIMA, E., KAKIZOE, T., SASAKI, H., SIGUMURA, T. & TERADA, M. (1992). Frequent association of p53 gene mutation in invasive bladder cancer. *Cancer Res.*, **52**, 1393–1398.

FULTS, D., BROCKMEYER, D., TULLOUS, M.W., PEDONE, C.A. & CAWTHON, R.M. (1992). p53 mutation and loss of heterozygosity on chromosomes 17 and 10 during human astrocytoma progression. *Cancer Res.*, **52**, 674–679.

HEALEY, J.H. & LANE, J.M. (1986). Chondrosarcoma. *Clin. Orthop.*, **204**, 119–129.

LANE, D.P. & CRAWFORD, L.V. (1979). T antigen is bound to a host protein in SV40-transformed cells. *Nature*, **278**, 261–263.

LEVINE, A., MOMAND, J. & FINLAY, C. (1991). The p53 tumor suppressor gene. *Nature*, **351**, 453–456.

MARKS, J.R., DAVIDOFF, A.M., KERNS, B.J., HUMPHREY, P.A., PENCE, J.C., DODGE, R.K., CLARKE-PEARSON, D.L., IGLEHART, J.D., BAST, Jr. R.C. & BERCHUCK, A. (1991). Overexpression and mutation of p53 in epithelial ovarian cancer. *Cancer Res.*, **51**, 2979–2984.

MASUDA, H., MILLER, C., KOEFFLER, H., BATTIFORA, H. & CLINE, M. (1987). Rearrangement of the p53 gene in human osteogenic sarcomas. *Proc. Natl Acad. Sci. USA*, **84**, 7716–7719.

MAZARS, R., SPINARDI, L., BENCHEIKH, M., SIMONY-LAFONTAINE, J., JEANTEUR, P. & THEILLET, C. (1992). p53 mutations occur in aggressive breast cancer. *Cancer Res.*, **52**, 3918–3923.

MILLER, C., ASLO, A., TSAY, C., SLAMON, D., ISHIZAKI, K., TOGUCHIDA, J., YAMAMURO, T., LAMPKIN, B. & KOEFFLER, H. (1990). Frequency and structure of p53 rearrangements in human osteosarcoma. *Cancer Res.*, **50**, 7950–7954.

MØRKVE, O. & LAERUM, O.D. (1991). Flow cytometric measurement of p53 protein expression and DNA content in paraffin-embedded tissue from bronchial carcinomas. *Cytometry*, **12**, 438–444.

MULLIGAN, L.M., MATLASHEWSKI, G.J., SCRABLE, H.J. & CAVENEE, W.K. (1990). Mechanisms of p53 loss in human sarcomas. *Proc. Natl Acad. Sci. USA*, **87**, 5863–5867.

NAKASHIMA, Y., UNNI, K.K., SHIVES, T.C., SWEE, R.G. & DAHLIN, D.C. (1986). Mesenchymal chondrosarcoma of bone and soft tissue. A review of 111 cases. *Cancer*, **57**, 2444–2453.

NIGRO, J.M., BAKER, S.J., PREISINGER, A.C., JESSUP, J.M., HOSTETTER, R., CLEARY, K., BINGER, S.H., DAVIDSON, N., BAYLIN, S., DEVILEE, P., GLOVER, T., COLLINS, F.S., WESTON, A., MODALI, R., HARRIS, C.C. & BOGELSTEIN, B. (1989). Mutations in p53 gene occur in diverse human tumor types. *Nature*, **342**, 705–707.

OLINER, J.D., KINZLER, K.W., MELTZER, P.S., GEORGE, D.L. & VOGELSTEIN, B. (1992). Amplification of a gene encoding a p53-associated protein in human sarcomas. *Nature*, **358**, 80–83.

PARADA, L.F., LAND, H., WEINBERG, R.A., WOLF, D. & ROTTER, V. (1984). Cooperation between gene coding p53 tumor antigen and *ras* in cellular transformation. *Nature*, **312**, 649–651.

RODRIGUES, N.R., ROWAN, A., SMITH, M.E.F., KERR, I.B., BODMER, W.F., GANNON, J.V. & LANE, D.P. (1990). p53 mutations in colorectal cancer. *Proc. Natl Acad. Sci. USA*, **87**, 7555–7559.

ROMANO, J.W., EHRHART, J.C., DUTHS, A., KIM, C.M., APPELLA, E. & MAY, P. (1989). Identification and characterization of a p53 gene mutation in a human osteosarcoma cell line. *Oncogene*, **4**, 1483–1488.

SAMESHIMA, Y., MATSUNO, Y., HIROHASHI, S., SHIMOSATO, Y., MIZOGUCHI, H., SUGIMURA, T., TERADA, M. & YOKOTA, J. (1992). Alterations of the p53 gene are common and critical events for the maintenance of malignant phenotypes in small-cell lung carcinoma. *Oncogene*, **7**, 451–457.

SOINI, Y., VÄHÄKANGAS, K., NUORVA, K., KAMEL, D., LANE, D.P. & PÄÄKKÖ, P. (1992). p53 immunohistochemistry in malignant fibrous histiocytomas and other mesenchymal tumors. *J. Pathol.*, **168**, 29–33.

STRATTON, M.R., MOSS, S., WARREN, W., PATTERSON, H., CLARK, J., FISHER, C., FLETCHER, C.D.M., BALL, A., THOMAS, M., GUSTERSON, B.A. & COOPER, C.S. (1990). Mutation of the p53 gene in human soft tissue sarcomas: association with abnormalities of the RB1 gene. *Oncogene*, **5**, 1297–1301.

TOGUCHIDA, J., YAMAGUCHI, T., RITCHIE, B., BEAUCHAMP, R.L., DAYTON, S.H., HERRERA, G.E., YAMAMURO, T., KOTOURA, Y., SASAKI, M.S., LITTLE, J.B., WEICHSELBAUM, R.R., ISHIZAKI, K. & YANDELL, D.W. (1992). Mutation spectrum of the p53 gene in bone and soft tissue sarcomas. *Cancer Res.*, **52**, 6194–6199.